# Microdissection tools to generate organoids for modeling the tumor immune microenvironment

**DOI:** 10.1038/s41378-024-00756-8

**Published:** 2024-09-10

**Authors:** Seth C. Cordts, Kanako Yuki, Maria F. Henao Echeverri, Balasubramanian Narasimhan, Calvin J. Kuo, Sindy K. Y. Tang

**Affiliations:** 1 Department of Mechanical Engineering, Stanford University, Stanford, CA USA; 2 Department of Medicine, Division of Hematology, Stanford University, Stanford, CA USA; 3 Department of Statistics, Stanford University, Stanford, USA

**Keywords:** Nanobiotechnology, Nanoscale devices

## Abstract

Patient-derived tumor organoids have emerged as promising models for predicting personalized drug responses in cancer therapy, but they typically lack immune components. Preserving the in vivo association between tumor cells and endogenous immune cells is critical for accurate testing of cancer immunotherapies. Mechanical dissection of tumor specimens into tumor fragments, as opposed to enzymatic digestion into single cells, is essential for maintaining these native tumor-immune cell spatial relationships. However, conventional mechanical dissection relying on manual mincing is time-consuming and irreproducible. This study describes two microdissection devices, the µDicer and µGrater, to facilitate the generation of intact tumor fragments from mouse B16 melanoma, a common model of human melanoma. The µDicer- and µGrater-cut tumor fragments were used to generate air‒liquid interface (ALI) organoids that copreserve tumor cells with infiltrating immune subsets without artificial reconstitution. The µDicer, consisting of a hexagonal array of silicon microblades, was employed to investigate the effect of organoid size. The viability of ALI organoid immune cells appeared insensitive to organoid sizes exceeding ~400 µm but diminished in organoids ~200 µm in size. The µGrater, consisting of an array of submillimeter holes in stainless steel, was employed to accelerate dissection. For the samples studied, the µGrater was 4.5 times faster than manual mincing. Compared with those generated by manual mincing, ALI organoids generated by the µGrater demonstrated similar viability, immune cell composition, and responses to anti-PD-1 immunotherapy. With further optimization, the µGrater holds potential for integration into clinical workflows to support the advancement of personalized cancer immunotherapy.

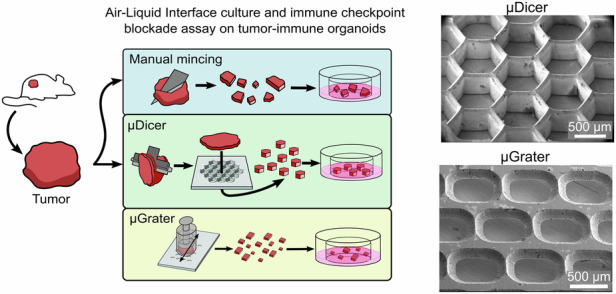

## Introduction

Cancer is the second leading cause of death in the US, with an estimated 1.9 million newly diagnosed cases in 2022 alone^1^. Genomics-guided cancer therapy has achieved limited success in predicting patient responses thus far, in part because cancer is not solely due to oncogenic mutations^2–4^. The tumor microenvironment (TME) is increasingly recognized to play a crucial role in tumorigenesis and the modulation of drug response^5,6^. An urgent need thus exists to establish high-fidelity human cancer models that can maintain the patient-specific TME and allow perturbation by drugs, immunotherapies, and/or other treatments, allowing the combination of genomics with phenotypic screening to predict therapeutic outcomes and accelerate personalized cancer therapy.

Immortalized 2D cancer cell lines and xenograft or transgenic animal models are common cancer models. Cell lines are limited in their ability to reproduce tumor-specific heterogeneity and pathophysiology^7^, whereas animal models are both costly and time-consuming to develop and apply. A promising alternative is patient-derived explants or organoids involving ex vivo culture of fresh tumor tissue^8–10^. These models have multiple advantages: they (1) resemble the source tumor phenotypically and genomically with patient-specific tumor heterogeneity^9–11^, (2) enable functional studies such as drug screening within a clinically actionable time frame^2^, and (3) can be generated from a wide range of tumor histologies^10,11^. Patient-derived organoids exhibit promise in advancing basic cancer research, as well as drug screening and biomarker discovery^11–19^, demonstrating the strong potential of patient-derived organoids as a clinically relevant model for personalized anticancer therapy.

Nevertheless, the lack of preservation of endogenous immune elements in organoids has been a major obstacle to modeling cancer immunotherapy. Prior studies have assembled organoids (or spheroids) from single tumor cells that lack stroma and cocultured them with peripheral immune populations^19–21^. However, these immune cells are phenotypically and functionally different from tumor-infiltrating lymphocytes (TILs)^22^, hindering investigations of cancer immunotherapy, such as immune checkpoint blockade^23^.

Recently, by combining patient-derived organoids with the air‒liquid interface (ALI) culture method, our group showed that tumor fragments could be grown as a cohesive entity that preserves both tumor cells and the endogenous stroma, including nonimmune elements (e.g., cancer-associated fibroblasts) and immune elements (T cells, B cells, NK cells, and macrophages)^11^. Importantly, ALI tumor organoids successfully recapitulated the immune diversity of the original tumor and immune checkpoint inhibition. Treatment with immune checkpoint inhibitors (anti-PD-1/PD-L1) enhanced human tumor-infiltrating lymphocyte (TIL) activation, expansion and tumor killing. These results demonstrate the promising utility of ALI organoids for modeling personalized immunotherapy. In contrast to ALI cultures, conventional submerged cultures do not preserve immune cells and fail to model immunotherapy.

Mechanical dissection of the tumor into submillimeter tissues, instead of enzymatic digestion into single cells, is critical for preserving the complex TME architecture of primary mouse or human tumors^11,24,25^. By avoiding single-cell dissociation, this method preserves native cell‒cell interactions to enable organoid formation and expansion^11^. Critically, mechanical dissection preserves the tumor parenchyma and stroma, including functional, tumor-specific TILs, and allows in vitro modeling of TME-intrinsic immune cell responses for cancer immunotherapy studies^11^.

Mechanical dissection of a fresh tumor may seem simplistic, but current methods for performing this task are suboptimal. The most common method is manual mincing with scissors or scalpels, which is time-consuming, ergonomically debilitating, and introduces contamination risk. Minced tissues are often large and nonuniform^26^, lack reproducibility from one operator to another, and can be difficult to standardize^11,13,14,24,27–29^. Indeed, this lack of standardization has been recognized as a key challenge in organoid technology^30–32^. Alternatively, semiautomatic mechanical tissue chopping has been used to dissect fresh tumors but requires time-consuming serial chopping and rotation in three dimensions^33^. In addition, this method often incompletely cuts through the depth of the tissue, and therefore requires subsequent manual separation and filtering. Tissue homogenizers pass input material through a mesh filter and/or shear with a rotating blade. However, these methods are typically intended to generate single cells rather than submillimeter tissue fragments^34,35^. As such, there is an unmet need for a more effective and standardized way to perform mechanical dissection of fresh tumors to generate microtissues within a size range that preserves the cellular contexture, endogenous immune cells, and function, thereby enabling organoid generation, downstream cancer immunology investigations, and patient-specific immunotherapy testing^11^.

**Fig. 1:**
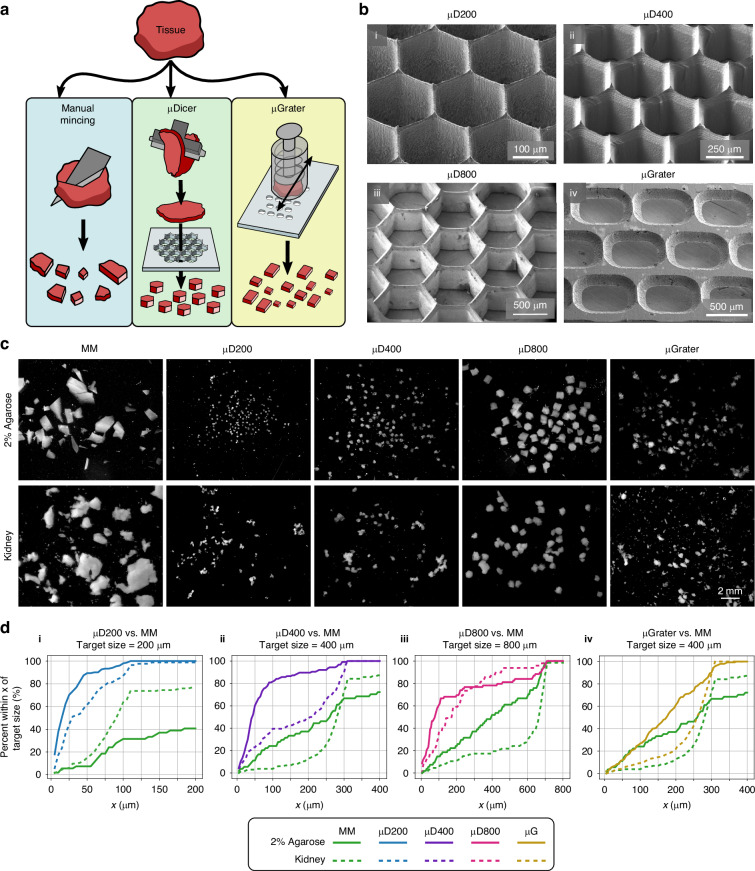
Overview of the µDicer and µGrater devices and initial testing with 2% agarose and porcine kidney tissue. **a** Diagram depicting the tissue dissection processes performed in this work. The conventional method, manual mincing (MM), was compared with the μDicer and μGrater methods. **b** SEM images of μDicers with blade spacings of (i) 200 μm, (ii) 400 μm, and (iii) 800 μm (“μD200”, “μD400”, and “μD800”, respectively). (iv) SEM image of the μGrater showing the backside where the stainless steel was etched to form sharp edges. **c** Dark field images of 2% agarose and porcine kidney after dissection by MM, μD200, μD400, μD800, and μGrater. The image contrast and brightness were enhanced to improve readability. **d** Empirical cumulative distribution function (ECDF) plots showing the percentage of microtissues within x μm of the target size, using images from “**c**” for 2% agarose and porcine kidney, comparing MM against (i) μD200, (ii) μD400, (iii) μD800, and (iv) μGrater. The size was compared according to the width of the minor axis of each microtissue. The target size was defined by the minimum spacing of the blades in the μDicer or μGrater. For example, “**d**(i)” shows that ~90% and ~10% of the agarose microfragments were within 50 μm of a target size of 200 μm, as generated by μD200 and MM, respectively

Here, we describe two microdevices, the µDicer and µGrater, for dissecting a fresh murine tumor into submillimeter tissue fragments, referred to as microtissues immediately after dissection and as organoids after culture. Although we have previously described the fabrication process of μDicer^36^, we have only tested the performance of the device with hydrogels and deceased tissues. A significant innovation and impact of the current study is the ability to dice live, fresh tumor tissues and maintain their viability after dicing. Using manual mincing as a benchmark, we compared (1) the viability immediately after µDicer and µGrater dissection and after culture at an air‒liquid interface (ALI), and (2) the response to treatment with immune checkpoint inhibitors (anti-PD-1) in ALI organoids generated via these three microdissection methods (Fig. 1a). We used ALI culture instead of conventional submerged culture, as it is directly pertinent to the immunotherapy modeling conducted in this study.

## Results and discussion

### Process flow of microtissue generation

The µDicer is a microscale dicing device consisting of a hollow hexagonal array of blades spaced hundreds of micrometers apart (Fig. 1bi–iii)^36^. A tissue slice, when pressed through the µDicer in a direction perpendicular to the plane of the device, is diced into many microtissues simultaneously. The µGrater is a microscale grater consisting of an array of microscale rectangular holes with sharp edges on all sides of the hole (Fig. 1biv). A bulk tissue, when pressed in directions both perpendicularly and tangentially to the plane of the µGrater, is grated into many microtissues simultaneously.

We used manual mincing (also referred to as “MM” in the figures) with scalpel blades as a benchmark because it is a common method for generating microtissues for ALI cultures and immunotherapy studies^11,24,25^. Compared with the μGrater and manual mincing, the µDicer allowed more precise control of the microtissue size in our preliminary tests with agarose and deceased porcine kidney (Fig. 1c, d). Consequently, we used µDicers with different blade spacings (200 µm, 400 µm, and 800 µm, respectively; referred to as “µD200”, µD400”, and µD800”) to determine the range of tumor organoid sizes that achieved viability similar to that generated by manual mincing. For the comparison between µDicer and manual mincing, we first sliced bulk tissue using a Compresstome before dicing or manual mincing. The thickness of the tissue slices was matched to the expected lateral dimensions of the microtissues, which were 200 µm, 400 µm, and 800 µm, corresponding to the blade spacing of the three µDicers tested. We fabricated the µDicers in silicon following our published method (see details in section “Fabrication of µDicers and adapters”, Note S1, and Fig. S2)^36^. The µDicers had a hexagonal pattern, instead of the square pattern used in prior work, to reduce the cut surface area-to-volume ratio and stress concentration at the corners. This pattern was expected to reduce cell damage and promote the overall viability of microtissues. The tip radius of the blades in the µDicers was approximately 0.9 μm^36^. We found that this sharpness was sufficient to dice through agarose, deceased porcine kidney, and B16 tumors. To dice the tissue after slicing, we used a 3 mm biopsy punch to collect a circular tissue slice and used the same biopsy punch to transfer the tissue slice above the µDicer. We then pressed the biopsy punch plunger to push the tissue slice through the µDicer. The biopsy punch plunger was modified to include a silicone tip to prevent damage to the µDicer blades.

For the comparison between the μGrater and manual mincing, the tissues were not presliced. Instead, bulk tissue specimens were grated or minced directly. The μGrater consisted of a two-dimensional array of rectangular holes (400 μm wide × 500 μm long, see Fig. S3) fabricated in a sheet of stainless steel (102 μm thick). Holes with sharp edges were formed by photochemical etching (see details in section “Fabrication of µGraters and adapters”). We chose stainless steel because it is biocompatible, easy to sterilize, and commonly used in surgical tools. Its fabrication is also compatible with commercial manufacturing process flow. With the current prototype, the edge tip radius was approximately 5 μm. Although it was possible to further sharpen the edge, we found that the current edges were sufficiently sharp to grate agarose, deceased porcine kidney, and B16 tumor while maintaining the robustness of the μGrater so that we could reuse the same device at least 5 times without a noticeable difference in the grating process. We did not monitor the robustness of the μGraters beyond 5 uses, but we anticipate that the devices to have capacity to endure additional uses. For grating, we loaded the bulk tissue into a custom μGrater tissue holder consisting of a spring-loaded plunger. While the tissue holder was held against the μGrater, we released the plunger to apply pressure on the tissue so that the tissue protruded partially through the rectangular holes on the device. We passed the tissue holder back and forth across the μGrater until all the tissue was grated. Compared with manual mincing, the μGrater was marginally better at producing more uniformly sized microtissues (Fig. 1c, d(iv)). However, unlike manual mincing, the μGrater did not generate microtissues > 1 mm.

### Comparison of organoids generated by the µDicer and manual mincing

**Fig. 2:**
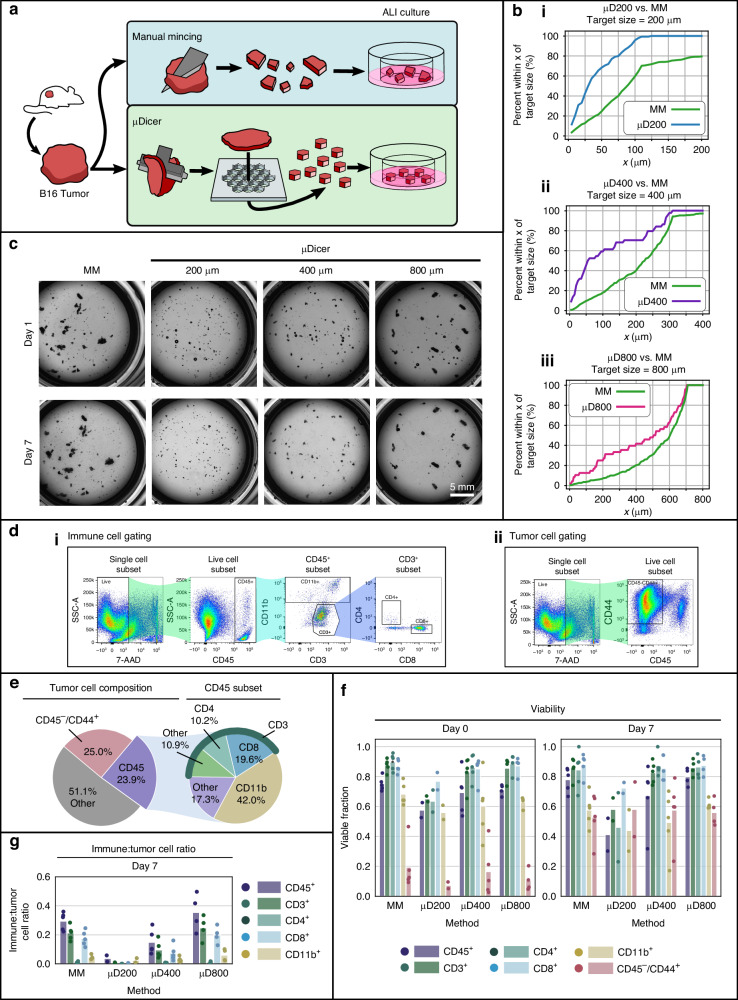
Size, cellular composition, and viability of B16 microtissues/organoids generated by the µDicer vs. MM at day 0 and day 7 of ALI culture. **a** Diagram depicting the tissue dissection processes performed to compare the conventional method, manual mincing (MM), with the μDicer. **b** Empirical cumulative distribution function plots comparing the size distribution of B16 microtissues generated by manual mincing (*N* = 312) to (i) μD200 (*N* = 139), (ii) μD400 (*N* = 44), and (iii) μD800 (*N* = 48) on Day 0. The size was compared according to the width of the minor axis of each microtissue. The target size was defined by the minimum blade spacing dimension of the μDicer. **c** Brightfield images from Day 1 and Day 7 of B16-MSIH organoid culture at the air‒liquid interface (ALI). The organoids were generated by MM, μD200, μD400, or μD800. **d** Flow cytometry gating strategy for representative B16-MSIH tumor organoids after 7 days of ALI culture for (i) tumor cells and (ii) immune cells. **e** Cellular composition of fresh B16-MSIH tumors plotted as the mean value of *N* = 5 independent tumors processed by manual mincing. **f** Viability of B16-MSIH tumor cells (CD45^−^/CD44^+^) and immune cells (CD45^+^, CD11b^+^, CD3^+^, CD4^+^, and CD8^+^) on Day 0 vs. Day 7 of ALI organoid culture after processing by MM, μD200, μD400, or μD800. We defined viability as the ratio of the viable cell count to the total cell count for a given cell type. Each data point corresponds to the viability of cells in microtissues/organoids generated by a given dissection method from one tumor sample. The height of the bars denotes the mean of the samples (*N* = 5 independent tumors for MM, μD400, and μD800; *N* = 2 for μD200). **g** Ratio of live immune cells (CD45^+^) to live B16-MSIH tumor cells (CD45^-^/CD44^+^) after 7 days of ALI organoid culture. Each data point corresponds to the viability of cells in microtissues/organoids generated by a given dissection method from one tumor sample. The height of the bars denotes the mean of the samples (*N* = 5 independent tumors for MM, μD400, and μD800; *N* = 2 for μD200)

**Fig. 3:**
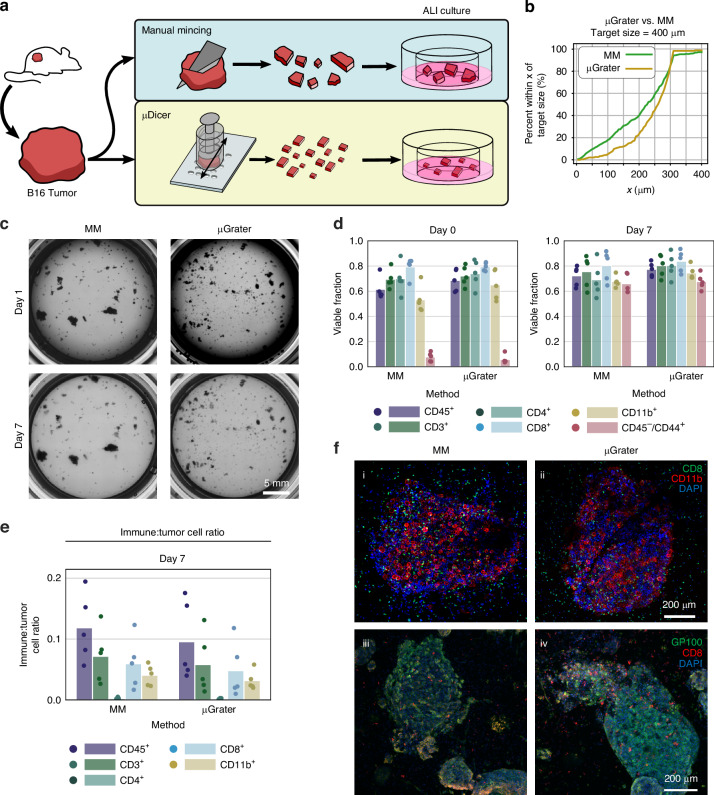
Size, cellular composition, and viability of B16 microtissues/organoids generated by the μGrater vs. MM at day 0 and day 7 of ALI culture. **a** Diagram depicting the tissue dissection processes performed to compare the conventional method, manual mincing (MM), with the μGrater. **b** Empirical cumulative distribution function (ECDF) plot comparing the size distributions of B16 microtissues generated by manual mincing (*N* = 312) and those generated using the μGrater (*N* = 231). The size was compared according to the width of the minor axis of each microtissue. The target size was defined by using the minimum blade spacing dimension of the μGrater. **c** Brightfield images from Day 1 and Day 7 of B16-MSIH organoid generation using MM and the μGrater in ALI culture. **d** Viability of B16-MSIH tumor cells (CD45^-^/CD44^+^) and immune cells (CD45^+^, CD11b^+^, CD3^+^, CD4^+^, and CD8^+^) on Day 0 vs. Day 7 after processing using MM and the μGrater. Each data point corresponds to the viability of cells in microtissues/organoids from one independent tumor sample. The height of the bars denotes the mean of these samples (*N* = 5 independent tumors). **e** Ratio of live immune cells (CD45^+^) to live B16-MSIH tumor cells (CD45^-^/CD44^+^) after 7 days of ALI organoid culture. Each data point corresponds to the viability of cells in microtissues/organoids from one tumor sample. The height of the bars denotes the mean of these samples (*N* = 5 independent tumors). **f** Immunofluorescence-stained images showing two different sets of images of cell composition within the organoids generated by (i, iii) MM and (ii, iv) μGrater after 7 days of ALI culture. (i, ii) CD8^+^ (green), CD11b^+^ (red), and DAPI (blue). (iii, iv) GP100^+^ (green), CD8^+^ (red), and DAPI (blue)

For the remainder of the study, we focused on organoids derived from a B16 melanoma mouse tumor, a commonly used model for human melanoma. An MSI-high variant of B16 was used to increase neoantigen burden^37^. B16 melanoma cells were implanted subcutaneously into syngeneic immunocompetent C57BL/6 mice to form tumors. Within 30 min of tumor resection, we generated microtissues by manual mincing with scalpel blades, µDicer (Fig. 2), or µGrater (Fig. 3).

Organoids were generated by manual mincing and µDicers with blade spacings of 200 µm, 400 µm, and 800 µm (Fig. 2a). Qualitatively, the largest organoids were generated by manual mincing, with a few exceeding 1 mm. The µDicer-generated organoids were closer to their intended size (Fig. 2b), i.e., the blade spacing of the respective µDicers used, compared with the organoids generated by manual mincing (also see Fig. S1). Organoids smaller than the intended sizes were also visible, likely due to the tissue handling steps (e.g., pipetting, suspension in gel), which could break some tissues into smaller fragments. Figure 2c shows microscopy images of the organoids in culture (see details in Note S1).

To determine the cellular subsets and their viability in the organoids, we followed published protocols^11,21,28^ and performed flow cytometry after the organoids were dissociated into single cells. We identified viable 7-AAD^-^ cells, CD45^+^ hematopoietic cells, infiltrating CD3^+^ T cells, CD4^+^ and CD8^+^ T cells, CD11b^+^ myeloid cells including macrophages, and CD45^-^/CD44^+^ tumor cells (Fig. 2d; also see Fig. S4 for a detailed gating scheme). A freshly minced tumor comprised ~25% tumor cells and small fractions (<5%) of tumor-infiltrating CD4^+^ and CD8^+^ T cells, with the remainder corresponding to other CD45^+^ immune subsets and/or presumed mesenchymal stroma (Fig. 2e).

Figures 2f and S5 show a comparison of the viability of different cell types in microtissues/organoids generated via different dissection methods on Day 0 (freshly dissected) and Day 7 of ALI culture, respectively. Here, and throughout the study, we defined viability as the ratio of viable cell count to total cell count for a given cell type in microtissues/organoids generated via the indicated method (see details in Note S1). Given the limited number of samples in this pilot study investigating the effect of the dissection method on viability, we restricted our main analysis to being descriptive in nature.

On both Day 0 and Day 7, the viability of all the cell types in the microtissues/organoids generated by µD400 and µD800 were similar to those generated by manual mincing. In contrast, the average viability of all cell types in microtissues/organoids generated by µD200 was lower than that generated by manual mincing. Comparing Day 0 vs. Day 7 for each dissection method, the viability of the tumor cells increased significantly, which was consistent with the aggressive nature of this tumor and the use of fetal bovine serum (FBS) in culture media favoring its growth in ALI culture. All immune cell types in the organoids generated by µD400, µD800, and manual mincing maintained viability after 7 days of ALI culture. In contrast, µD200 processing decreased all immune cell types in the organoids, as evidenced by a reduced average viability after ALI culture (Figs. 2f and S5) and a decreased ratio of live immune cells to live tumor cells (Fig. S6). Consistent with prior work^11^, the ratio of immune cells to tumor cells, i.e., the level of tumor-infiltrating immune cells, generally decreased with time after ALI culture, but the level of infiltrating immune cells was lowest in µD200 organoids (Fig. 2g).

These results were surprising because, in previous work, organoids larger than ~200–500 µm were typically less viable, which was attributed to an inadequate nutrient supply and suboptimal oxygenation^28,38–41^. Culture in oxygen-permeable devices has been shown to improve organoid viability^42^. Here, we attributed the maintenance of viability in organoids generated by µD400, µD800, and manual mincing to the high level of oxygenation in ALI cultures^27^. The reduced viability of microtissues generated by µD200 on Day 0 was potentially due to the larger cutting surface area-to-volume ratio and the corresponding mechanical stress imposed on the cells when dicing with µD200 compared with µD400 and µD800. Furthermore, the size of µD200-generated ALI organoids might be too small to preserve the TME, with insufficient stromal cells to support the viability of immune cells in B16 tumors.

The detailed mechanisms by which organoid size affects the viability of different cell types warrant further investigations. Nevertheless, our results have important practical implications. Under the conditions tested, the viability of important cell types, including key TIL populations, appeared insensitive to organoid size larger than ~400 µm. These findings raised the possibility of using alternative microdissection methods that might sacrifice some precision on organoid size but would allow more rapid and facile tumor processing than the µDicers.

### Organoids generated by the µGrater and manual mincing had comparable viability and composition

A challenge in using the µDicer is the requirement for tumor preslicing prior to dicing. To overcome this challenge, we developed the µGrater to generate organoids from bulk tumors directly without any preprocessing steps (Fig. 3a). Because the viability of microtissues and organoids larger than 400 µm was insensitive to their size, we chose a µGrater hole width of 400 µm. We found that the exact shape of the holes (rectangular, circular, angled slots) did not impact the performance of the µGrater. Unlike manual mincing, the µGrater prevented the generation of large (exceeding millimeter size) microtissues (Fig. 3b, c). Additionally, microtissue generation from the same bulk tumor took approximately 20 s when the µGrater was used vs. 1.5 min when manual mincing was used. This 4.5x improvement in speed is expected to be significant for processing more tumors or tumors of larger sizes.

Figure 3d shows that the viability of all cell types in microtissues/organoids generated by the µGrater was similar to that generated by manual mincing (also see p values of comparisons in Table S1). After 7 days of ALI culture, the ratio of live immune cells to live tumor cells was similar in the organoids generated via both methods (Fig. 3e). Immunohistochemical staining of sections from the organoids generated via both methods revealed similar histologies and confirmed the presence of tumor-infiltrating CD8^+^ T cells and CD11b^+^ myeloid cells (Fig. 3f, see Note S1 for the detailed immunofluorescence imaging methods).

### PD-1 immune checkpoint blockade expanded CD8^+^ TILs and induced tumor killing to similar levels in organoids generated by the µGrater and manual mincing

Despite the similar viability of tumor and immune cells from organoids generated by µD400, µD800, and µGrater compared with manual mincing, we focused subsequent comparisons on µGrater vs. manual mincing given the ease of use and lack of pre-processing steps for the µGrater.

**Fig. 4:**
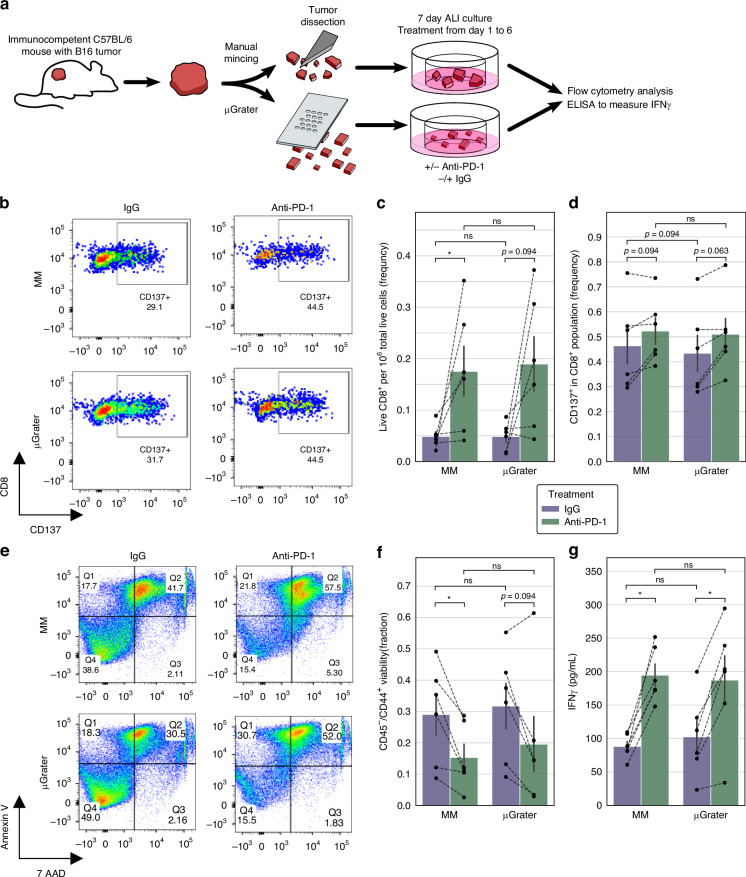
Evaluating TIL function and response to PD-1 blockade in B16 organoids generated by the μGrater vs. MM. **a** Diagram of the experimental design. Minced or μGrater-processed B16 tumor tissues were plated in ALI and cultured for 7 days. IgG or anti-PD-1 treatment started on Day 1. Flow cytometry and ELISA analyses were performed on Day 7. **b** Representative flow cytometry plots showing the gating strategy for determining the percentage of CD137^+^ cells in the CD8^+^ cell population on Day 7 of B16-MSIH ALI organoid culture after treatment with anti-PD-1 vs. control IgG. **c** Live CD8^+^ cells per 10^6^ live cells on Day 7 of B16-MSIH ALI organoid culture after treatment with anti-PD-1 vs. control IgG. **d** Fraction of live CD137^+^ cells in the live CD8^+^ cell population on Day 7 of B16-MSIH ALI organoid culture after treatment with anti-PD-1 vs. control IgG. **e** Representative flow cytometry plots showing the gating strategy for live tumor cells by Annexin V and 7-AAD staining in the C45^−^/CD44^+^ cell population on Day 7 of B16-MSIH ALI organoid culture after treatment with anti-PD-1 vs. control IgG. **f** Viability of CD45^-^/CD44^+^ tumor cells in B16-MSIH ALI organoid cultures on Day 7 after treatment with anti-PD-1 vs. control IgG. Live cells were determined by the Annexin V^-^/7-AAD^-^ cell population. **g** Secreted IFNγ concentrations in conditioned medium were analyzed by ELISA on Day 7 of B16-MSIH ALI organoid culture after treatment with anti-PD-1 vs. control IgG. For **c, d, f, g**, the dashed lines connect paired data points for organoids originating from the same tumor sample. The height of the bars denotes the mean, and the black lines denote the standard error of these samples (*N*=6 independent tumors). IgG treatment and anti-PD-1 treatment of organoids generated by MM vs. μGrater were compared using the Wilcoxon signed-rank test. Comparisons where the difference was statistically significant (*p* < 0.05) are marked with an asterisk (*), while those not reaching statistical significance (*p* > 0.1) are denoted as ‘n.s.’ (not significant). For cases where 0.05 < *p* < 0.1, the exact *p* value is provided for clarity

Next, we evaluated TIL function and response to PD-1 blockade in ALI B16 melanoma organoids created by manual mincing vs. µGrater. We treated the organoids with function-blocking recombinant monoclonal anti-murine PD-1 antibodies or control IgG for 6 days (Fig. 4a and Note S1). Anti-PD-1 therapy increased the average number of CD8^+^ TILs per total live cells (Fig. 4c), paralleling CD8^+^ TIL expansion by anti-PD-1 therapy within B16 tumors in vivo^23,43,44^. Anti-PD-1 treatment activated CD8^+^ T cells, as indicated by an increase in the number of CD137^+^/CD8^+^ TILs (Fig. 4b, d)^45^. The level of activation was similar in the organoids generated by the µGrater and manual mincing. Importantly, anti-PD-1 therapy promoted tumor cell killing in organoids generated by µGrater and manual mincing, with an increase in both early apoptotic cells (annexin-V^+^/7-AAD^-^) and late apoptotic and necrotic cells (annexin-V^+^/7-AAD^+^) compared with the control using IgG (Fig. 4e). The reduction in the proportion of viable (annexin-V^-^/7-AAD^-^) tumor cells after anti-PD-1 treatment was similar between the organoids generated by the µGrater and manual mincing (Fig. 4f and Table S1). Consistent with these results, anti-PD-1 therapy increased the organoid secretion of IFNγ into the conditioned medium to similar levels in the organoids generated using both methods (Fig. 4g and Table S1). All comparisons between the µGrater and manual mincing using the Wilcoxon signed rank test were insignificant (also see Table S1). Although some comparisons between anti-PD-1 and IgG treatments revealed a *p* value > 0.05 for manual mincing and/or the µGrater, trends consistent with anti-PD-1-induced tumor killing, T-cell activation and expansion were generally observed (Fig. 4c, d, f, g) and could be refined by further studies. Notably, similar trends were observed in the anti-PD-1 treatment of organoids generated by µD400 and µD800 (Fig. S7).

## Conclusions

We have described mechanical microdissection technologies to facilitate the generation of tumor organoids that preserve tumor cells *en bloc* with endogenous immune cells, including tumor-reactive TILs, to enable in vitro modeling of PD-1 blockade immunotherapy. Manual mincing is currently the most common method for producing such organoids from fresh tumors. Thus far, most microtechnologies developed for organoid research have aimed to facilitate organoid formation from single cells or streamline organoid culture and drug screening^46^. The key innovation of our work is the creation of microdissection devices to perform parallel dicing or grating of a fresh tumor into many viable submillimeter tissues. The µDicer generation of microtissues with a narrower size range than manual mincing enabled interrogation of the effect of organoid size on the viability of the constituent cells. While prior work has examined the consequences of varying the size of spheroids assembled from single cells, such effects have been more challenging to study in organoids generated by mechanical dissection, given the difficulties associated with controlling organoid size using conventional dissection methods, i.e., manual mincing^13,27–29,32^. Although it was possible to perform manual mincing followed by filtering to obtain microtissues of the desired sizes, the size distribution can vary between operators or experiments, introducing significant variation in the yield of organoids of the desired sizes. Notably, the microtissue/organoid sizes generated by the µDicer from B16 tumors were not as uniform as those from agarose or deceased porcine kidney, likely due to the mechanical properties of fresh B16 tumors and our tissue handling steps, which broke some B16 microtissues/organoids into smaller pieces after dissection by the µDicers. We do not believe these small pieces impacted our results because all of the organoids (typically a few hundred) were pooled in a well to assess viability and the anti-PD-1 response, with the small pieces constituting a small percentage of the cells in the well (Fig. S1). However, a better way to transfer and distribute microtissues/organoids is needed, for example, for large screens where each well may have only a few organoids.

A major finding of our study is that for the B16 mouse tumor model used, the viability of important cell types appeared relatively insensitive to organoid sizes exceeding ~400 µm. However, cell viability decreased in organoids of ~200 µm. This result contrasts with prior work that used manual mincing with limited collagenase digestion of similar mouse tumor models to generate short-term tumor spheroids. This prior study revealed that spheroids > 100 μm, 40–100 μm, and <40 μm cultured in a 3D microfluidic device displayed insignificant differences in live immune populations^28^. It is possible that differences in the specific tumor model and culture conditions led to differences in our observations on the effect of organoid size. In particular, the high level of oxygenation in the ALI culture used in this study^26^ allowed very large (~1 mm) organoids generated by manual mincing to remain viable. In liquid cultures in multiwell plates commonly used for large drug screens, we anticipate that organoid viability and drug response will be more sensitive to the organoid size. In this case, we anticipate that tighter microtissue size control by the µDicer or an improved version of the µGrater (see discussions below) will lead to better microtissue viability and drug response than manual mincing. However, liquid culture testing requires separate optimization of growth media and culture conditions and is unfortunately beyond the scope of the current paper. Finally, while larger organoids are often thought to have a greater likelihood of hypoxia, cells have a significant capacity to adapt metabolic rates to nutrient and oxygen supply conditions^41^. Further studies are necessary to better understand the effects of organoid size on cellular metabolism and viability.

Practically, our findings prompted the development of the µGrater, the reduced precision in microtissue size was offset by enhanced speed and ease of use compared with those of the µDicer. The µGrater generated microtissues with viability and immune cell function matching those generated by manual mincing in ALI culture. For the tumor samples in this study, µGrater was 4.5x faster than manual mincing, not only reducing the processing time but also limiting ergonomic exhaustion and contamination risk. We anticipate that a further increase in grating speed is possible without compromising the viability of the microtissues. If validated, we expect the µGrater to be a more scalable approach than the µDicer (which requires preslicing of the tumor specimen) and manual mincing in generating a large quantity of organoids. This aspect is the subject of an ongoing investigation. The limited number of independent tumor specimens tested in the current study did not allow attainment of full statistical significance for several immune function endpoints in µGrater-processed organoids. To fully validate the utility of the µGrater for generating organoids, larger studies involving additional tumor specimens will be necessary in future work. In the current design of the μGrater tissue holder, a simple spring was used to apply pressure to push the tumor so that it protruded partially through the μGrater holes while a transverse motion was applied to grate the tumor. An inherent limitation of this design was that the pressure applied by the spring to the tissue decreased as the tissue was grated and the spring extended. This design likely contributed to the heterogeneity in microtissue size caused by the µGrater. The current work involves improving the holder design to ensure that the pressure applied to the tumor remains constant throughout the grating process. With this improvement, we expect to improve the size uniformity of microtissues generated by the µGrater.

Ongoing work includes developing a better understanding of the physics of the grating of biological tissues, increasing the blade sharpness, incorporating automation elements, and validating the method in other tumors. To preserve the viability of endogenous cell types, other tumors and/or culture methods may require a tighter size range than that provided by the μGrater. In this case, the μDicer could find utility, although investigation is necessary to further streamline the dicing process flow.

As the range of therapies and combination trials continues to expand, there is an unmet need for better tumor processing and organoid generation technologies for preclinical and clinical use. We believe that our µGrater technology has the potential to benefit personalized cancer immunotherapy by increasing the speed and yield of organoid generation while retaining the complex TME and functional endogenous immune elements. These capabilities can increase the utility of valuable tumor specimens from patients for downstream drug screening and the prediction of individualized patient responses to therapies. Upon further validation, optimization, and automation, our µGrater technology could become standardized and has substantial potential for incorporation into clinical workflows and adoption into a rigorous Clinical Laboratory Improvement Amendments (CLIA) laboratory environment. While this work focused on the response to immune checkpoint inhibition, our microdissection methods could also facilitate assays of other anticancer modalities, such as chemotherapy or radiation therapy, or extension to treatments for nonneoplastic disorders.

## Methods

### Fabrication of µDicers and adapters

We fabricated µDicers following our published method with slight modifications^36^ Fig. S2 shows the fabrication process and parameters. See Note S1 for details.

We sterilized the µDicers in 70% isopropanol for 30 min and then air-dried them. Before use, the µDicers were coated in 3% F68 solution (Thermo Fisher Scientific, J66087-36) for 1 hour to reduce the friction between the tissue and the µDicer surface. The µDicers were then rinsed in PBS two times, dried with compressed air, and stored dry until use. These sterilization steps and F68 coating steps were repeated for each round of experiments.

A tissue dicing adapter was designed in AutoCAD Fusion 360 and printed on Formlabs Form 2 using biomedical clear resin. The adapter comprised 2 pieces that were clamped together to hold the μDicer in place between two silicone gaskets (Fig. S8). In some experiments, the adapter was modified to include a 3 mm inner diameter (I.D.) bushing inserted into the adapter to act as a vertical guide when pressing the tissue through the μDicer. A 3 mm stainless steel dowel with a 3 mm silicone tip was used to press the tissue through the μDicer. This modification was made to improve the pressure uniformity.

### Fabrication of µGraters and adapters

The μGrater was fabricated in stainless steel using a photochemical etching process performed by Switzer Manufacturing (New York, USA). Briefly, a two-dimensional μGrater design with rectangular holes was drawn in AutoCAD (see Fig. S3) to pattern a photoresist mask on the top and bottom sides of a 0.004” sheet of 304 stainless steel. The sheet was chemically etched from both sides isotropically to form a sharp edge where the etch fronts met. After etching, the photomasks were stripped from the stainless steel sheets before they were electropolished to improve the sharpness and surface finish. The µGraters were used without any coating because we did not have any issues with tissue adhesion.

To clamp the μGrater during use, we 3D printed a frame (Fig. S3c) on Formlabs Form 2 using biomedical amber resin. The frame was designed to clamp the thin μGrater in tension. Without applying tension, the μGrater would deflect and hinder grating. A custom μGrater tissue holder was used to press the tissue against the μGrater during grating (Fig. S3d). The holder consisted of a stainless-steel standoff as the barrel, a wing-bolt and nylon standoff as the plunger, and a spring to apply the compressive force against the tumor. The spring was chosen to prevent the tumor from being overly compressed (in which case the tumor tissue would squeeze through the μGrater holes) or from being under compressed (in which case the tissue would not grate at all)^47^. The characterization of the μGrater (e.g., the effect of grating mechanics vs. edge tip radius, hole size, and protrusion depth) is complex and beyond the scope of this study focused on the application of the μGrater toward the generation of tumor organoids. The detailed characterization of the μGrater is the subject of an ongoing study.

### Syngeneic murine tumor model

*Msh2*-deficient MSI-high B16F10 melanoma cells (referred to as B16-MSI-high cells)^48^ were generously provided by Dr. Timothy Chan (MSKCC). B16-MSI-high cells were cultured in RPMI 1640 with 10% FBS and Normocin. Female C57BL/6 J mice were purchased from The Jackson Laboratory. Six- to nine-week-old mice were subcutaneously implanted with 5 × 10^5^ B16-MSI-high cells. The animals were maintained on a 12-hour light/dark cycle in a temperature- and humidity-controlled room with food and water.

### Operation of µDicers

For comparison between manual mincing and μDicer, the tumor was first sliced using a tissue slicer (Compresstome VF-310-0Z, Precisionary Instruments, Massachusetts USA) before dicing or manual mincing. Fresh tumor samples were resected from the mice, washed with PBS, and transferred to culture medium (10% FBS RPMI 1640 supplemented with 10% FBS, GlutaMAX (Gibco), 1× nonessential amino acids (Gibco), 1× sodium pyruvate (Gibco), 1× penicillin–streptomycin, 1× Normocin and 1× insulin/selenium/transferrin cocktail (Gibco)) on ice until use (<30 min).

The tumors were first sliced into 800-, 400-, and 200-µm-thick slices using a Compresstome. Briefly, the tissue was mounted onto a Compresstome plunger and embedded in 2% low-melt agarose. The collection bath was filled with ice-cold Hank’s balanced salt solution (HBSS) without calcium or magnesium (HBSS-). Alternate slices were used for manual mincing and the µDicer to reduce variation due to intratumor heterogeneity. As the slices were generated, they were manually sorted using a small spatula into Petri dishes on ice in HBSS- buffer before dicing.

We sterilized the tissue dicing adapter by soaking it in 70% isopropyl alcohol for 30 min and then allowed it to dry in the tissue culture hood. The adapter, gaskets, and μDicer were assembled in the tissue culture hood immediately before use.

The tissue slices were cut with a 3-mm-diameter biopsy punch. The biopsy punch plunger was modified to include a silicone tip to prevent damage to the μDicer blades. The tissue slice was diced by pressing the biopsy punch plunger manually. The tissue was pressed until it could be seen coming out of the backside of the µDicer. The tissues were extruded through the backside of the μDicer and collected in a Petri dish on ice by gently pipetting HBSS-containing medium on the backside of the μDicer. The same μDicer was used for repeated samples unless they broke and needed to be replaced. Typically, 5–10 slices were diced for each condition depending on the initial tumor size.

### Operation of µGraters

For the comparison between manual mincing and the μGrater, the tumors were not sliced. Bulk tumor samples were manually minced or grated directly. Fresh tumor samples were resected from the mice and placed in the aforementioned culture medium on ice until use (<30 min). All the μGraters and reusable parts were sterilized by autoclaving at 121 °C for 30 min or disinfected by soaking in 70% isopropanol for 30 min and then left to dry in a cell culture hood.

At the start of the experiment, a sterile μGrater was held over a Petri dish using the 3D printed frame (Fig. S3). The tumor was loaded into a custom μGrater tissue holder by retracting the spring-loaded plunger and placing the tumor into the barrel using sterile tweezers. The μGrater tissue holder was then inverted so that the tissue faced down on the μGrater. While the tissue holder was manually held against the μGrater, the plunger was released to apply pressure on the tissue. The holder was manually passed back and forth across the μGrater until all the tissue was grated. Once complete, ice-cold HBSS buffer was used to wash away any tissue stuck to the bottom of the μGrater by surface tension to collect into the Petri dish. See Video S1 for a demonstration of tumor grating using the μGrater.

### Manual mincing

As a benchmark for the μDicer and μGrater, we performed manual mincing of the tumor. For comparison with μDicer, we manually minced tumor slices generated from the Compresstome. Slices were collected into a Petri dish on ice using tweezers to minimize fluid transfer. The tissue was minced using two scalpels in a chopping and slicing motion repeatedly for 1 min 30 s. For comparison with the μGrater, the tumor was divided into 2 pieces and not sliced prior to manual mincing. The tissue was minced using two scalpels repeatedly for 1 min 30 s. After mincing, the tissue was resuspended in ice-cold culture media.

### Characterizing size distribution

To quantify the size distribution of microtissues generated by the μDicer, the μGrater, and manual mincing, we dissected 2% agarose LE and deceased porcine kidney tissue. The 2% agarose LE and porcine kidney were processed as described above to dissect the samples into microtissues. After dissection, the microtissues were filtered through a 40 µm filter to mimic the removal of single cells. Microtissues > 40 µm were collected in a Petri dish for imaging. We obtained dark field images of the 2% agarose LE and kidney microtissues using a digital microscope (STEMI 508, Zeiss). The images were analyzed in FIJI by autolocal thresholding to identify the microtissue regions of interest. Manual segmenting was performed to correct any touching microtissues that were incorrectly segmented by autothresholding. Any overlapping microtissues that could not be segmented were excluded from the analysis. Empty Petri dishes filled with PBS were imaged to identify the background noise that could be eliminated from analysis. We used the “analyze particles” tool in FIJI to quantify the size of the microtissues in each image. Since the microtissues were in a disordered arrangement in the Petri dish and some tissue slices were thicker than the nominal size of the µDicer holes, the width of the minor axis was used as the dimension for comparison to avoid bias in the analysis based on the thickness of the tissue slice before dicing. The data are plotted in Fig. 1d for 2% agar and porcine kidney as an empirical cumulative distribution function (ECDF)^49^ calculated as follows:

$$F\,(x)=\frac{1}{n}\mathop{\sum }\limits_{i=1}^{n}{\rm{I}}({x}_{i}\le x)$$

$${x}_{i}=\left|{w}_{i}-{w}_{o}\right|$$

where *w*_*i*_ is the width of the minor axis of an ellipse fit to a given microtissue, *w*_0_ is the target width, *n* is the total number of microtissues, and I is the indicator function equal to 1 when ${x}_{i}\le x$ and equal to 0 otherwise. The same approach was used to characterize the size distribution of the B16 microtissues.

### Statistical analysis

Because of the small number of samples, we used descriptive statistics (means) to compare the viability of different cell types in microtissues/organoids generated by manual mincing vs. µDicer (Fig. 2) and by manual mincing vs. µGrater (Fig. 3). Although the number of samples was small, we compared the viability of different cell types in manual mincing vs. μGrater using the Mann‒Whitney test for completeness (Table S1). We compared the metrics of IgG treatment and anti-PD-1 treatment of organoids generated by manual mincing vs. μGrater using the Wilcoxon signed rank test (Fig. 4 and Table S1).

## Supplementary information

Supplementary Material

SI Video1

## Data Availability

The data used in this manuscript are available from the corresponding author upon reasonable request.
