# Supplementary material for: Microdissection tools to generate organoids for modeling the tumor immune microenvironment
Source: Microsyst Nanoeng. 2024 Sep 10;10:126. doi: 10.1038/s41378-024-00756-8 (PMC11385579; doi:10.1038/s41378-024-00756-8)
Supplement: Supplementary file 1 — Supplementary Material [file 41378_2024_756_MOESM1_ESM.docx]

**Microdissection tools to generate organoids for modeling the tumor immune microenvironment**

Seth C. Cordts *^a^*^†^, Kanako Yuki *^b^*^†^, Maria Fernanda Henao Echeverri*^b^*, Balasubramanian Narasimhan*^c^*, Calvin J. Kuo*^b^*, Sindy K.Y. Tang*^a^**

*^a^ Department of Mechanical Engineering, Stanford University*

*^b^ Department of Medicine, Division of Hematology, Stanford University*

*^c^ Department of Statistics, Stanford University*

[*sindy@stanford.edu](mailto:*sindy@stanford.edu)

^†^ These authors contributed equally to this work.

**Supplementary information**

**Note S1. Additional information on experimental methods.**

**Fabrication of µDicers in silicon.**

We grew a silicon oxide layer as an etch stop at the back of a single crystal silicon device wafer. We spin-coated CrystalBond™ (Electron Microscopy Sciences) onto a second silicon wafer which served as a carrier wafer. We then bonded the device wafer on top of the carrier wafer. To form an array of microblades (step 1), we used a single etch mask composed of silicon dioxide and performed tapered etches in the device wafer by cycling between isotropic etches (for t_1-iso_ seconds) and deep reactive-ion etches (DRIE) using a Bosch process (for n_1_ cycles). We repeated this process N rounds in an inductively coupled plasma deep silicon etcher (PlasmaTherm, Versaline). We chose the values of t_1-iso_ and n_1_ to control the angle of the overall etch and, thus, the half angle of the blade. For some devices, we replaced the tapered etch with isotropic etch only and were able to obtain blade tips that were sufficiently sharp for cutting tissues. The number of rounds (N) controlled the point at which the etch fronts undercutting the oxide mask met to form the blades. We chose N = 4 so that the etch stopped immediately before the etch fronts met. N>4 resulted in over-etching and instability of the mask. In step 2, we used the Bosch process (n_2_ cycles) to etch the through-holes through the silicon, stopping at the silicon dioxide layer used as an etch stop deposited at the backside of the silicon wafer. The depth of the etch was measured with digital microscopy at intermittent points. In step 3, we sharpened the blades with an isotropic etch (t_3-iso_). We determined the value of t_3-iso_ by monitoring the progression of the etch fronts every 100 s until the corner tips formed. After completing the etches, we released the device wafer from the carrier wafer and removed the top oxide mask and back oxide. Individual µDicers were then released by separating them from each other using tweezers along etched trenches between each µDicer.

**Air-liquid interface (ALI) tumor organoid culture.**

Tumors with a 10 ± 2 mm diameter were dissected, washed with PBS, and transferred to the aforementioned culture medium on ice before processing by the μDicer, the μGrater, or manual mincing. Fresh tumors were briefly checked by FACS and the immune cold tumors were excluded from the experiments. During tissue handling steps, small fragments and single cells were unintentionally created. The tissue suspension was passed through a 40 μm filter and rinsed with 1 mL of HBSS- buffer 5 times. Any cells or clusters smaller than 40 μm were filtered out. Tissues > 40 μm were then resuspended in culture medium. These tumor microtissues were then centrifuged at 600 x g for 3 minutes or left to settled to the bottom of a 15 mL conical tube for 10 minutes and then the medium was aspirated carefully. The microtissue were gently mixed with collagen gel and plated in ALI as described before^1,2^. Briefly, culture inserts with a permeable 0.4 μm pore membrane (PICM03050, Millicell-CM, Millipore) were put in 6-well culture plates. 1 mL of collagen gel (Cellmatrix type I-A, Nitta gelatin) was added to the insert as a bottom layer gel without tissue. After solidification of the bottom layer, 1 mL of tissues/collagen gel mixture was layered on the top of the bottom layer. 1.5 mL of culture medium with the addition of 30 U/mL IL-2, 0.5 ng/mL IL-7, 10 ng/mL IL-15 and 50 μM 2-ME, hereinafter referred to as CM+ was added into the wells outside of the inserts^3^. Organoid images in the insert were taken with a Keyence microscope (BZ-X710).

We did not quantify the number of cells per microtissue because, unlike spheroids that are assembled from single cells with known seeding cell type and density, our microtissues were dissected directly from the tumor which had a mixture of cell types and extracellular matrix (ECM). Nevertheless, we can approximate the number of cells per microtissue by assuming each cell is ~20 μm across. For the μD200 microtissues, if we assume the height, width, and length of each microtissue to be 200 μm, and without taking into account the packing efficiency or ECM, there were approximately 1,000 cells. Similarly, there were approximately 8,000, and 64,000 cells in each μD400 and μD800 microtissue, respectively.

**Flow cytometry analysis.**

Microtissues or organoids in a well were pooled and then dissociated into single cells before flow cytometry analysis following published protocol^2^. Briefly, collagen gels were digested with collagenase type 4 (LS004210, Worthington), followed by neutralization with FBS and then incubated with Liberase (05401020001, Roche) followed by neutralization with FBS to make single cells. Cells were washed and resuspended in flow cytometry buffer (3%FBS in PBS). Then, cells were stained with Fc block (#101320, BioLegend) and the fluorescent conjugated antibodies (BV421 anti-CD45 antibody: 30-F1 #103134, BioLegend; BV786 anti-CD3e antibody: 145-2C11 #564379, BioLegend; BV650 anti-CD11b antibody: #101259, BioLegend; PECy7 anti-CD44 antibody: IM7 #103030, BioLegend; APC anti-CD4 antibody: #100412, BioLegend; PerCP-Cy5.5 anti-CD8a antibody: 53-6.7 #551162, BioLegend; PE anti-CD137 antibody: 17B5 #106106, BioLegend), followed by staining of 7-AAD (A1310, Invitrogen)/Annexin V-APC (#640920, BioLegend). Stained cells were analyzed by a BD FACSAria II (BD Biosciences) and FlowJo software (BD Life Sciences). Cells were first gated on forward scatter/side scatter and then gated on single cells. Live cells were separated by 7-AAD staining. These were further gated for CD45^+^, followed by CD11b^+^ and CD3^+^, then CD3^+^ was separated to CD4^+^ and CD8^+^ subsets. The CD45^-^CD44^+^ population was identified as B16-MSIH tumor cells. For cell viability analysis of each subset, live cell gating followed each subset gating.

**Whole-mount immunofluorescence staining.**

Whole-mount immunofluorescence staining was performed as described before^4^. After 7 days culture, organoid-embedded collagen gels were washed with PBS for 10 minutes twice and fixed with 4% paraformaldehyde (PFA) in PBS for 1 h at room temperature. After washing with PBS for 10 minutes twice, the PFA was quenched with PBS containing 100 mM glycine for 30 minutes at room temperature with gentle rocking. Organoids were blocked/permeabilized with 10% donkey serum in staining buffer (PBS containing 0.5% Tween-20, 0.1% BSA and 0.05% NaN_3_) for 2 hours at room temperature with gentle rocking, followed by incubation with primary antibodies (anti-melanoma GP100 antibody: 1:200, #ab137078, Abcam) in PBS containing 10% donkey serum, 0.5% Tween-20 and 0.1% BSA for 2 days at room temperature. Organoid-embedded collagen gels were then washed with staining buffer for 30 minutes 4 times at room temperature and incubated with secondary antibodies, fluorescence-conjugate primary antibodies and DAPI for overnight at room temperature in staining buffer (Alexa Fluor 488-donkey anti-rabbit IgG: 711-545-152, Jackson ImmunoResearch; Alexa Fluor 555-anti-CD8 antibody: 1:100, ab280863, Abcam; Alexa Fluor 647 anti-CD11b antibody: 1:200, ab197702, Abcam). Following additional washes with staining buffer for 30 minutes 6 times, PBS for 30 minutes and deionized water for 15 minutes, organoid-embedded collagen gels were transferred to the slides and covered with the mounting medium (Vectashield, Vector Laboratories) and the coverslips for imaging using a Zeiss LSM 900 confocal microscope.

**Assay for anti-PD-1-dependent TIL activation within organoids.**

Organoid cultures were established as above and supplemented with CM+ containing 20 μg/mL anti-PD-1 (CD279) antibody (RMP1-14: #114110, BioLegend) or 20 μg/mL isotype control IgG (RTK2758: #400544, BioLegend) on day 1 for 6 days. On the sixth day of treatment (seventh day of culture), the organoids were dissociated into single cells and stained for flow cytometry analysis as described above.

**IFNγ enzyme-linked immunosorbent assay (ELISA).**

Conditioned media were collected on day 7 from ALI culture. IFN-γ concentration in the media was measured by ELISA MAX Deluxe Set Mouse IFN-γ (#430804, BioLegend) according to the manufacturer’s instructions.

**Figure S1. Weighted histograms as an alternative to quantify the size distribution of microtissues of B16 tumor generated by manual mincing vs. μDicer vs. μGrater.**

We present an alternative way to quantify the size distribution of B16 microtissues by using weighted histograms. The weighted histogram is defined as follows:

$$P_{j}=\frac{\sum_{w_{i}\epsilon B_{j}} A_{i}}{\sum_{i} A_{i}}$$

$$B_{j}=[25(j-1)+min \left( w \right), 25j+min\left( w \right)]$$

Where $P_{j}$ is the area fraction (bar height) for the $j^{th}$ bin, $w$ is a list of the widths for the minor axis of all microtissues, $w_{i}$ is the minor axis width of the $i^{th}$ microtissue, $B_{j}$ is the range of the $j^{th}$ bin, and $A_{i}$ is the area of the $i^{th}$ microtissue. A bin size of 25 μm was chosen for the histograms.

Conceptually, the weighted histogram shows the proportion of microtissues by area in each bin of the histogram sorted by minor axis width. The rationale for using the weighted histogram instead of a histogram is based on the practical application of MM, μDicer, and μGrater and our method of measuring viability, which is by pooling all organoids in a dish and then dissociating them into single cells for flow cytometry analysis. When generating microtissues to grow into organoids, the ideal scenario is to generate uniformly sized microtissues. However, for all three methods, small pieces of tissue are unintentionally generated (<100 microns) largely due to the tissue handling steps after dissection. The weighted histogram evaluates the dissection performance by putting emphasis on the area of microtissues that fall within each minor width size range. This approach differs from a histogram, which presents the counts of microtissues in various size bins, giving equal weight to each microtissue regardless of its size. Large microtissues have more cells than small microtissues, making area-based assessment more representative of the number of cells within microtissues of a given size range than counts in a histogram.

Weighted histograms for B16 tumor microtissues generated by a(i-iii) μDicer, a(iv) μGrater, and a(v-vii) manual mincing (MM) on day 0. Images in (a) were used for analysis in Fig. 2b and Fig. 3b. Any object with area < 1600 µm^2^ (~40 µm across) was assumed to be small debris and excluded from analysis. The data for MM (b(v)) was combined from 3 separate experiments (corresponding to images a(v-vii)). The count of microtissues for MM, μD200, μD400, μD800, and μGrater were N = 658, N = 209, N = 46, N = 55, and N = 657, respectively.

**
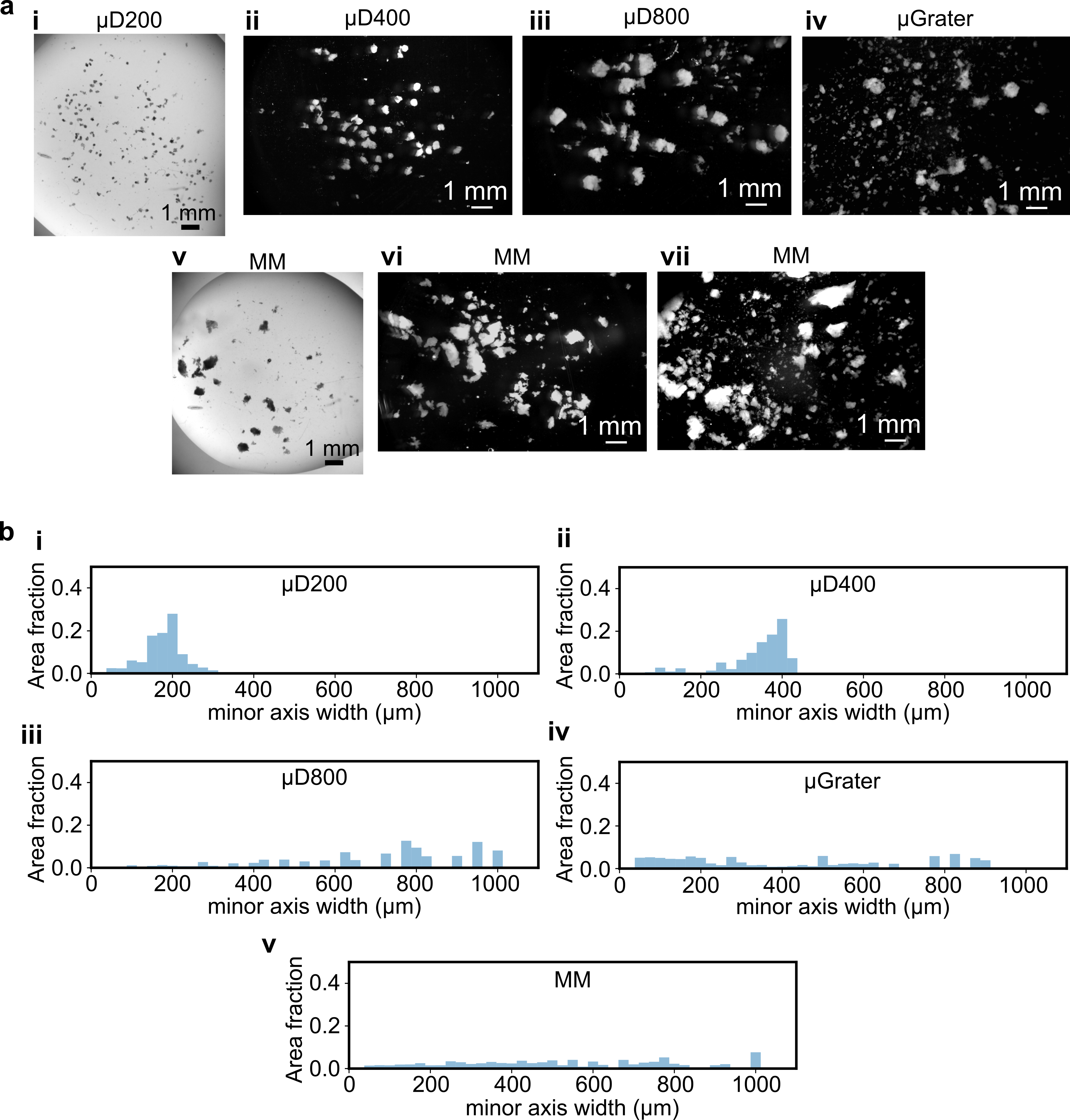
**

**Figure S2. μDicer Fabrication method.**

a) The modified fabrication method adapted from^5^. b) Mask designs for the μDicers with blade spacings of 200 μm, 400 μm, and 800 μm, respectively. c) The etch parameters for the plasma etching steps.


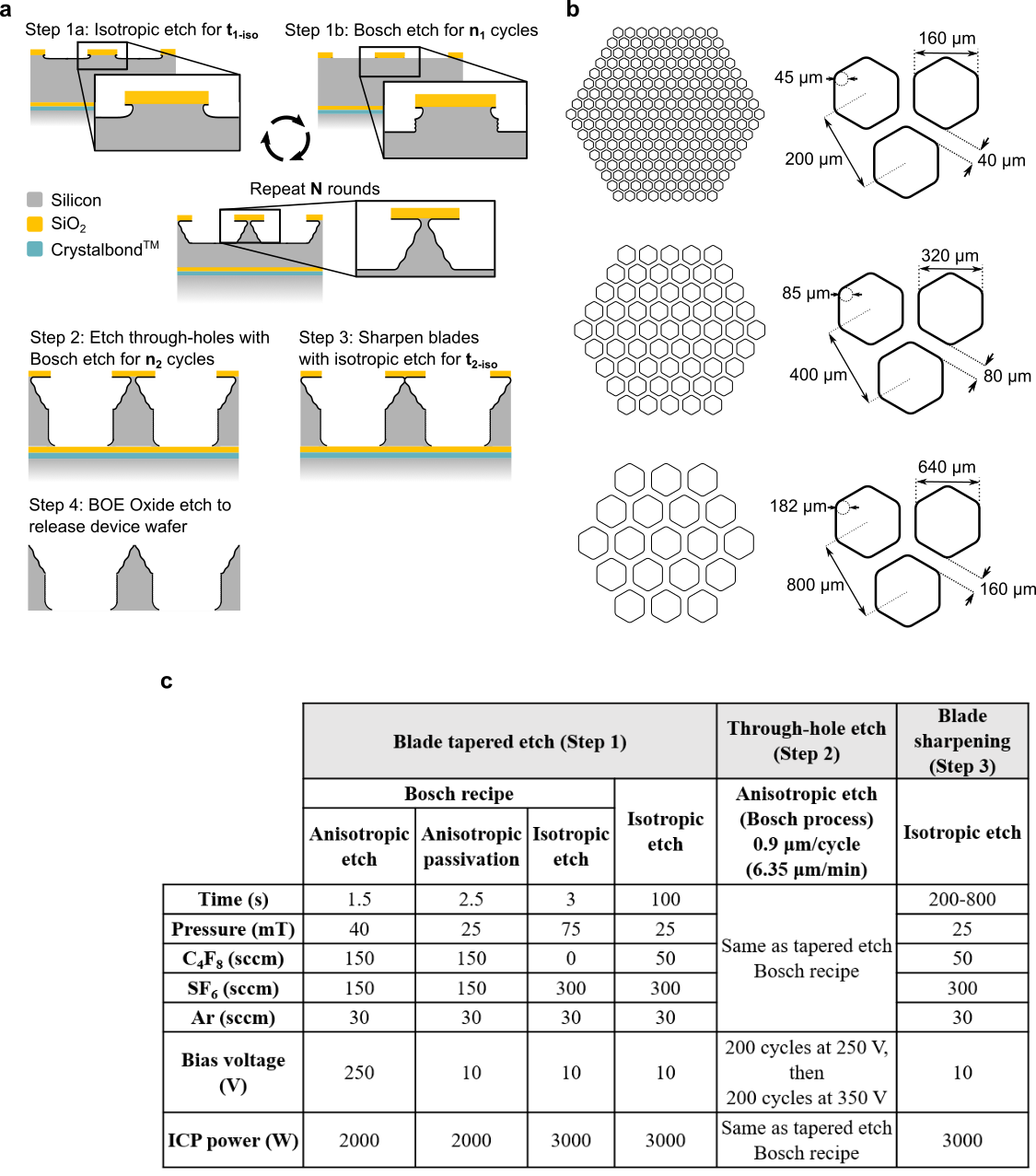


**Figure S3. μGrater design, fabrication, and assembly.**

a) The μGrater mask design consists of an array of rectangular holes which form the blades after chemical etching. The perimeter holes form the mounting points of the device on the adapter.

b) Fabrication of μGrater. Photochemical etching of stainless steel is an established commercial process. Therefore, we had used a company (Switzer Manufacturing, New York, USA) to perform the etching of our devices based on the mask design we sent them. We requested the use of 0.004” stainless steel based on the limits of their etching process in minimum feature size (≥110% of material thickness), and minimum corner radius (≥ material thickness). We also requested the use of 304 stainless steel due to its biocompatibility and that it is easier to etch than 316 stainless steel.^6^ The diagram illustrates a typical fabrication process for one hole in the μGrater.

Because the detailed fabrication is proprietary to the company, we can only describe the overall concept of the process flow here with the information we were able to obtain from the company. A two-dimensional μGrater design (see part a) was used to pattern a photoresist mask on the top and bottom sides of a 0.004” sheet of 304 stainless steel. E-9200 series dry film resist (Eternal Technology Corp., Virginia, USA) was used as photoresist. A potassium carbonate-based solution with proprietary additives was used as the developer. The sheet was chemically spray-etched from both sides isotropically using a ferric chloride-based etchant to form a sharp edge where the etch fronts met.^7^ After etching, the photomasks were stripped from the stainless steel sheets using a sodium hydroxide-based solution with proprietary additives. After that, the sheet was electropolished by applying DC power to the parts in a mixture of sulfuric acid and phosphoric acid as the electrolyte. Electropolishing was used to improve the sharpness and surface finish.

c) The 3D printed adapter consists of a 2-part frame connected by a 2-bar linkage that serves as a clamp to easily lock the μGrater in tension when in use, while also making it easy to remove.

d) A cross-section schematic diagram of the custom-made tissue plunger consisting of a ¼” inner diameter stainless-steel standoff for the barrel of the plunger, a wing bolt for the plunger handle, a ¼” outer diameter standoff which is epoxied into place to contain the spring in the plunger, and a threaded nylon dowel which serves as the plunger tip. The spring used in all experiments in this study had a spring constant of k = 0.16 N/mm.

e) A photo of the custom-made tissue plunger.


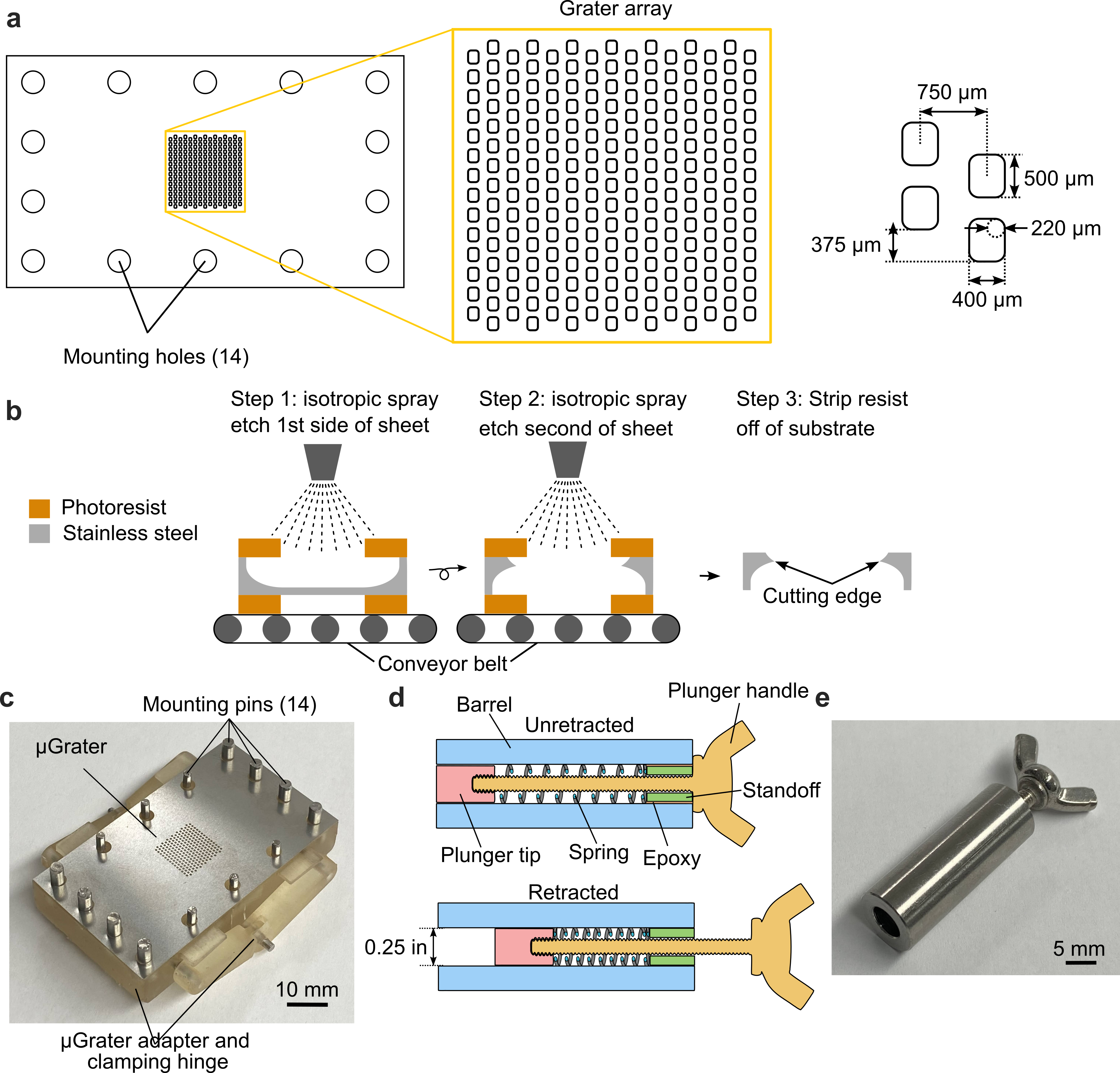


**Figure S4. Gating scheme for flow cytometry data.**

The microtissues or organoids were dissociated into single cells. Cell suspensions were first gated by FSC-A vs. SSC-A, and gated for single cells by FSC-W vs. FSC-H, and SSC-W vs. SSC-H. Then, cells were gated for viability by 7-AAD staining, followed by cell subtype-specific staining (CD45, CD3, CD11b, CD8, CD4, CD44).

**
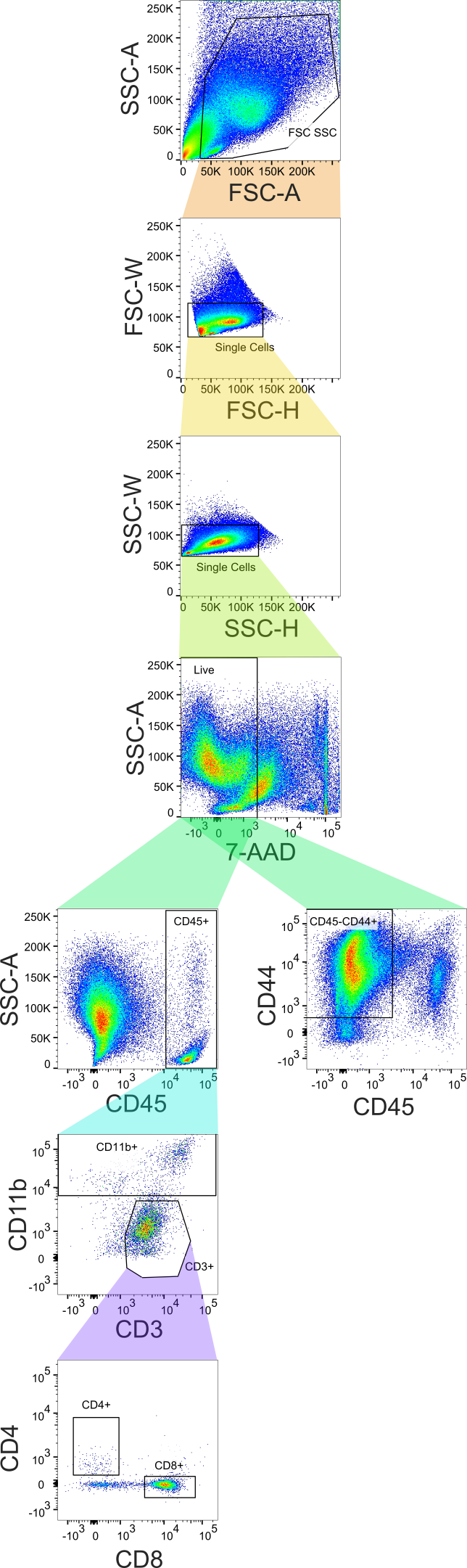
**

**Figure S5. Cell viability compared by processing method.**

Viability of each cell type from microtissues/organoids generated by a) MM, μD200, μD400, and μD800 and b) MM and μGrater. The lines connect data points from the same tumor sample.


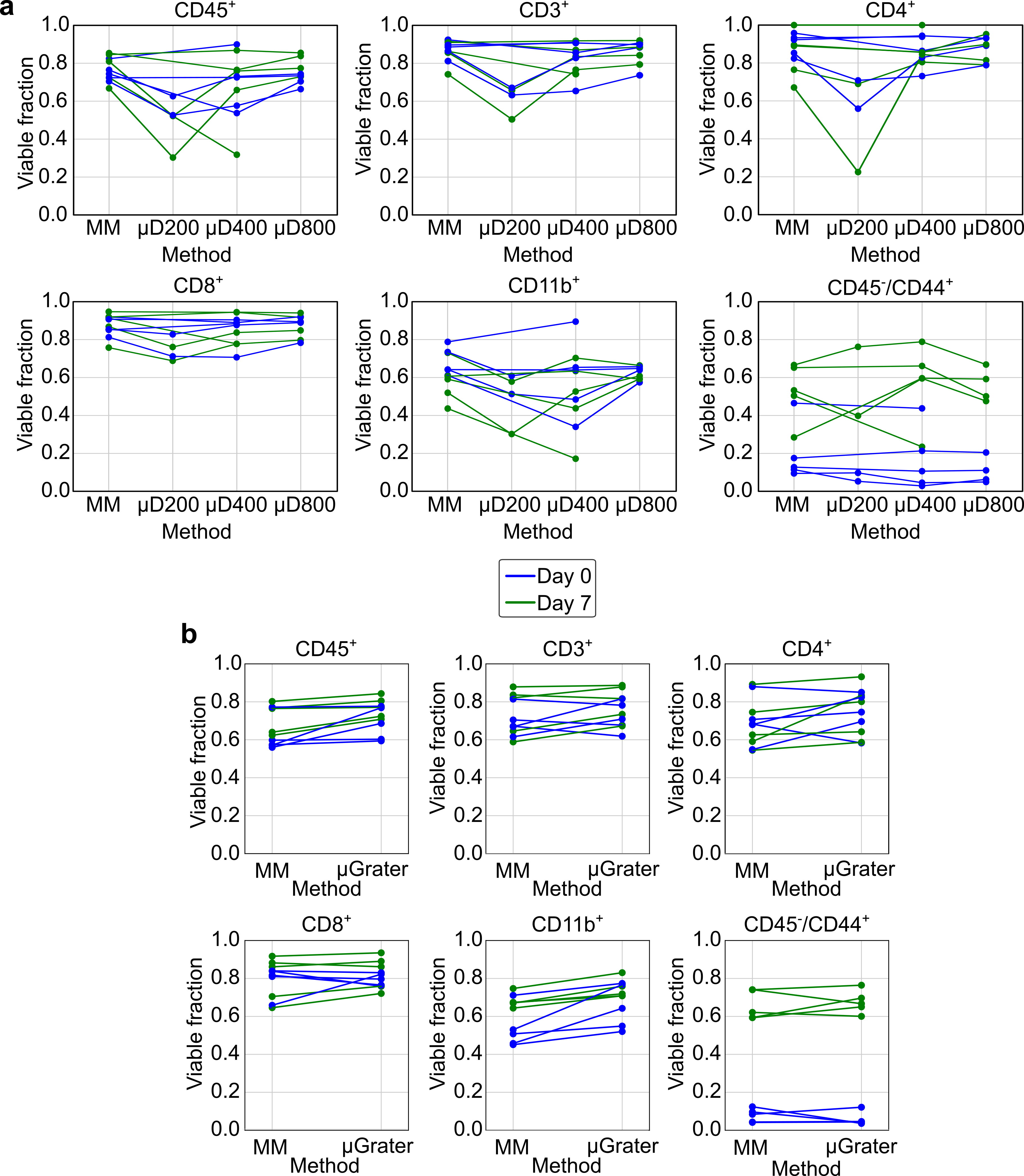


**Figure S6. Ratio of live immune cells to live tumor cells.**

The ratio of live immune cells (CD45^+^) to live tumor cells (CD45^-^/CD44^+^) from microtissues/organoids generated by MM vs. μD200, MM vs. μD400, MM vs. μD800, and MM vs. μGrater.


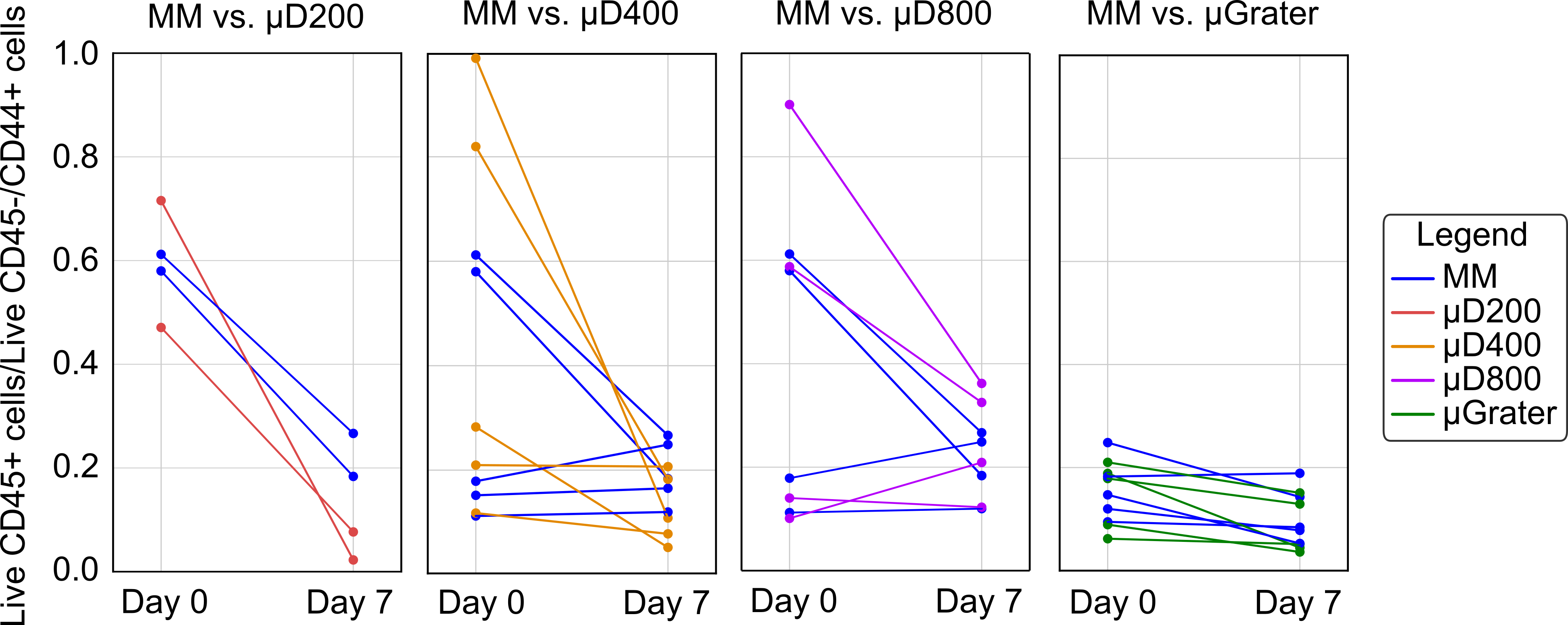


**Figure S7. Anti-PD-1 treatment of organoids generated by MM, µD400, and µD800.**

a) Viability of CD45^-^/CD44^+^ tumor cells in B16-MSIH ALI organoid culture on day 7 after treatment with anti-PD-1 vs. control IgG. Live cells were determined by the Annexin V^-^/7-AAD^-^ cell population. b) Fraction of live CD137^+^ cells in the live CD8^+^ cell population on day 7 of B16-MSIH ALI organoid culture after treatment with anti-PD-1 vs. control IgG.


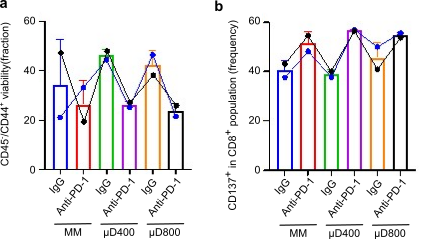


**Figure S8. μDicer adapter.**

a) 3D printed adapter design for the μDicer. b) Images of the adapter disassembled and assembled with a μDicer.


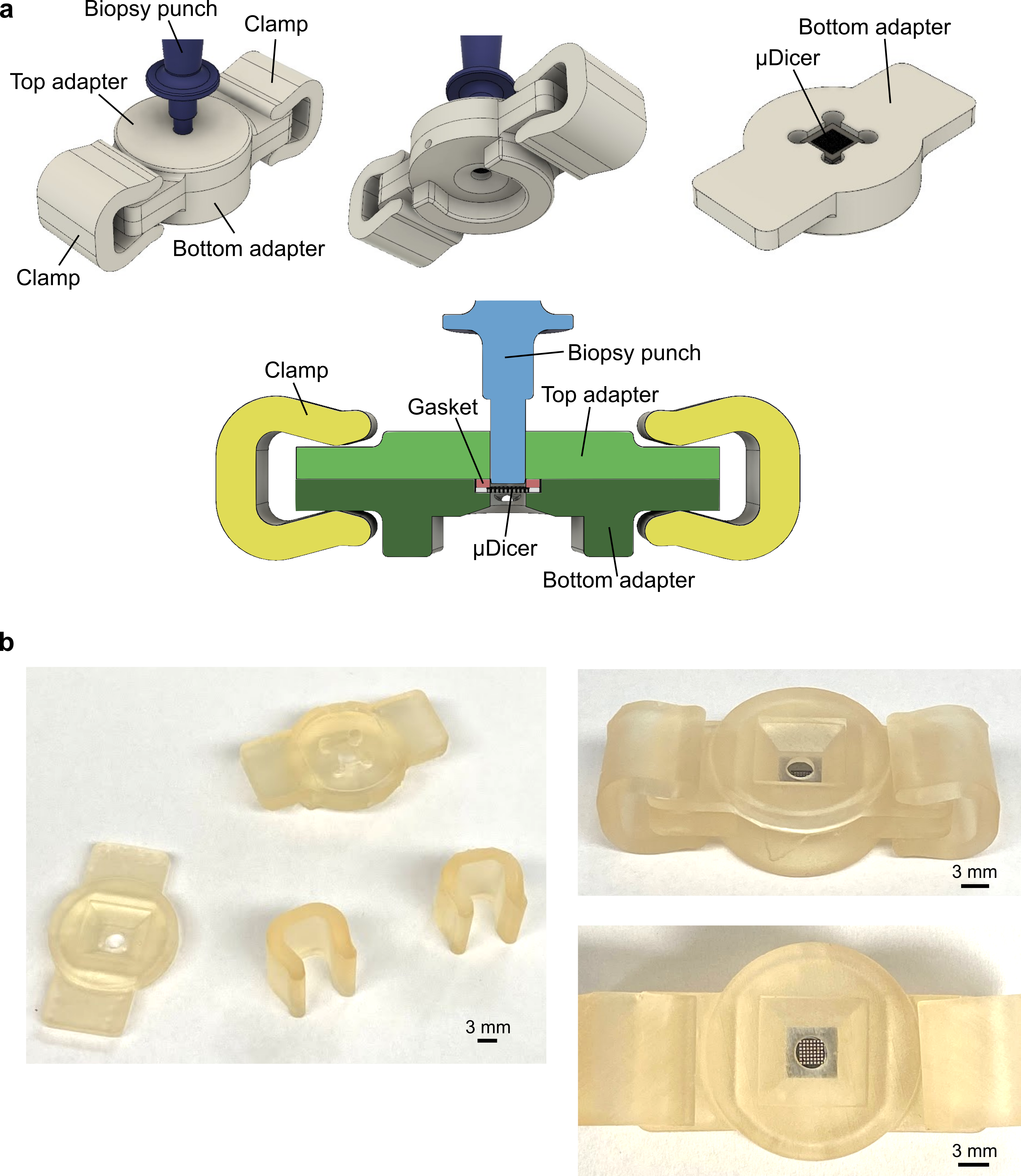


**Table S1. p values comparing MM vs μG**

Although the number of samples were few, we compared manual mincing (MM) vs μGrater (denoted as μG below) using the Mann-Whitney test for completeness, in microtissues on day 0 and in ALI organoids from N=5 independent tumors. There were no significant differences between the viability of cells between microtissues/organoids generated by MM vs. μGrater for both day 0 and day 7.

| Cell type | CD45^+^ | CD3^+^ | CD4^+^ | CD8^+^ | CD11^+^ | CD45^-^/CD44^+^ |
| --- | --- | --- | --- | --- | --- | --- |
| MM vs μG (N=5) | 0.151 | 0.421 | 0.548 | 0.548 | 0.095 | 0.421 |

p values for % live cells for different cell types on day 0 (for Fig. 3d):

| Cell type | CD45^+^ | CD3^+^ | CD4^+^ | CD8^+^ | CD11^+^ | CD45^-^/CD44^+^ |
| --- | --- | --- | --- | --- | --- | --- |
| MM vs μG (N=5) | 0.310 | 0.548 | 0.151 | 0.548 | 0.056 | 0.548 |

p values for % live cells for different cell types after 7 days of ALI culture (for Fig. 3d):

IgG treatment and anti-PD-1 treatment of organoids generated by MM vs. μGrater were compared using the Wilcoxon signed rank test in ALI organoids from N=6 independent tumors. No significant difference was found between MM vs. μGrater for any treatment measurements.

MM vs. μG p values for Fig. 4c, d, f, g:

|  | Live CD8^+^ per 10^6^ live total cells | Proportion of live CD137^+^ cells in the live CD8^+^ cell population | Viability of CD45^-^/CD44^+^ cells | IFNγ concentration |
| --- | --- | --- | --- | --- |
| MM vs μG  IgG  (N=6) | 0.125 | 0.094 | 0.438 | 0.688 |
| MM vs μG  anti-PD-1  (N=6) | 0.875 | 0.563 | 0.688 | 0.438 |

|  | Live CD8^+^ per 10^6^ live total cells | Proportion of live CD137^+^ cells in the live CD8^+^ cell population | Viability of CD45^-^/CD44^+^  cells | IFNγ concentration |
| --- | --- | --- | --- | --- |
| IgG vs anti-PD-1  MM  (N=6) | 0.031 | 0.094 | 0.031 | 0.031 |
| Anti-PD-1 vs IgG  μG  (N=6) | 0.094 | 0.063 | 0.094 | 0.031 |

IgG vs. anti-PD-1 treatment p values for Fig. 4c, d, f, g:

**Video S1.**

Demonstration of the μGrater. The μGrater is preloaded onto the 3D-printed adapter and placed in a 100 mm Petri dish on ice. The tumor sample is placed, with tweezers, into the barrel of the tissue plunger while the plunger handle is retracted. The tissue plunger is flipped over and held firmly against the μGrater while the plunger handle is released. The tissue plunger is moved back-and-forth over the μGrater until all the tissue has been grated. The microtissues are collected into the 100 mm Petri dish by pipetting buffer over the backside of the μGrater.

References

1. Li, X. *et al.* Oncogenic transformation of diverse gastrointestinal tissues in primary organoid culture. *Nat. Med.* **20,** 769–777 (2014).

2. Neal, J. T. *et al.* Organoid modeling of the tumor immune microenvironment. *Cell* **175,** 1972–1988.e16 (2018).

3. Wagar, L. E. *et al.* Modeling human adaptive immune responses with tonsil organoids. *Nat. Med.* **27,** 125–135 (2021).

4. Salahudeen, A. A. *et al.* Progenitor identification and SARS-CoV-2 infection in human distal lung organoids. *Nature* **588,** 670–675 (2020).

5. Cordts, S. C., Castaño, N., Koppaka, S. & Tang, S. K. Y. Fabrication of a silicon *μ* Dicer for uniform microdissection of tissue samples. *Appl. Phys. Lett.* **119,** 011904 (2021).

6. Li, L., Breedveld, V. & Hess, D. W. Creation of superhydrophobic stainless steel surfaces by acid treatments and hydrophobic film deposition. *ACS Appl. Mater. Interfaces* **4,** 4549–4556 (2012).

7. Nageswara Rao, P. & Kunzru, D. Fabrication of microchannels on stainless steel by wet chemical etching. *J. Micromech. Microeng.* **17,** N99–N106 (2007).
